# Therapeutic apheresis: An effective strategy for a combined targeting of circulating lipoproteins, inflammatory markers, PFAS, and microplastics in cardiometabolic and neurodegenerative disease?

**DOI:** 10.61373/bh026a.0024

**Published:** 2026-08-04

**Authors:** Stefan R. Bornstein, Waldemar Kanczkowski, Romy Walther, Romy Kronstein-Wiedemann, Dieter Fischer, Ioannis Oikonomakos, Roman N. Rodionov, Sergey Tselmin, Yannick P. Kok, Philip A. Mavberg, Jordi Petriz, Michael D. Ward, Yandy Marx Castillo-Aleman, Carlos Schuster, Michael Petegorsky, Constanze Hantel, Felix Beuschlein, Giatgen A. Spinas, Torsten Wuestefeld, Saravana K. Ramasamy, Yusuf Ali, Joseph J. Y. Sung, Torsten Tonn, Mario Siervo, Andrew Aswani, Mahmoud Barbir, Ludwig Aigner, Henning Morawietz, Rinkoo Dalan, Kaomei Guan, Ma-Li Wong, Julio Licinio, Charlotte Steenblock

**Affiliations:** 1 Department of Internal Medicine III, University Hospital Carl Gustav Carus, Technische Universität Dresden, 01307 Dresden, Germany;; 2 School of Cardiovascular and Metabolic Medicine and Sciences, Faculty of Life Sciences & Medicine, King’s College London, London, SE5 9NU UK;; 3 Department of Endocrinology, Diabetology and Clinical Nutrition, University Hospital Zurich (USZ) and University of Zurich (UZH), 8091 Zurich, Switzerland;; 4 Laboratory for Experimental Transfusion Medicine, Transfusion Medicine, Faculty of Medicine Carl Gustav Carus, Technische Universität Dresden, 01307 Dresden, Germany;; 5 Institute for Transfusion Medicine, German Red Cross Blood Donation Service North-East, 01307 Dresden, Germany;; 6 Leibniz-Institut für Polymerforschung Dresden e.V., 01069 Dresden, Germany;; 7 Ayus Laboratories AG, 8810 Horgen, Switzerland;; 8 Ayus Medical Basel, Ayus Medical Group AG, 4051 Basel, Switzerland;; 9 Fundació Institut d’Investigació en Ciències de la Salut Germans Trias i Pujol (IGTP), 08916 Badalona, Spain;; 10 Thermo Fisher Scientific, Fort Collins, CO 80524, USA;; 11 Abu Dhabi Stem Cells Center (ADSCC), Abu Dhabi, UAE;; 12 Yas Clinic Khalifa City (YCKC) Hospital, Abu Dhabi, UAE;; 13 Proxima Health, Inc., Austin, TX 78701, USA;; 14 Lee Kong Chian School of Medicine, Nanyang Technological University, 636921 Singapore;; 15 Clinical Research Unit, Khoo Teck Puat Hospital, 768828 Singapore;; 16 Institute for Transfusion Medicine and Immunohematology, Goethe University Hospital Medical School, 60590 Frankfurt, Germany;; 17 German Red Cross Blood Donor Service Baden-Wuerttemberg-Hessen, 60528 Frankfurt, Germany;; 18 School of Population Health, Curtin University, Bentley, WA 6102, Australia;; 19 Dementia Centre of Excellence, enAble Institute, Curtin University, Bentley, WA 6102, Australia;; 20 Department of Intensive Care Medicine, Guy’s and St Thomas’ NHS Foundation Trust, SE1 7EH London, UK;; 21 Santersus AG, 8001 Zurich, Switzerland;; 22 Cardiology Department, Royal Brompton & Harefield Hospitals, part of Guy’s and St Thomas’ NHS Foundation Trust, Harefield, UB9 6JH UK;; 23 Institute of Molecular Regenerative Medicine, Paracelsus Medical Private University, 5020 Salzburg, Austria;; 24 KLARIS Precision Health Research Clinic, Tan Tock Seng Hospital, National Healthcare Group, 308433 Singapore;; 25 Institute of Pharmacology and Toxicology, Technische Universität Dresden, 01307 Dresden, Germany;; 26 Department of Psychiatry and Behavioral Sciences, State University of New York Upstate Medical University, Syracuse, NY 13210, USA.

**Keywords:** Microplastic, nanoplastic, PFAS, apheresis, lipoproteins

## Abstract

Lipoprotein apheresis is a highly effective and well-established extracorporeal therapy designed to remove lipoproteins from human circulation on a large scale, particularly in patients with progressive cardiovascular diseases when conventional treatments fail to achieve adequate lipoprotein reduction. Beyond its lipid-lowering capabilities, therapeutic apheresis is used to eliminate pathogenic substances, such as autoantibodies and immune complexes, and to exert immunomodulatory effects. Recent research highlights its potential to reduce inflammatory mediators, making it a promising intervention for conditions associated with systemic inflammation, including neurodegenerative diseases and post-infectious syndromes. In addition to its established applications, therapeutic apheresis is being explored as a novel approach to address emerging environmental health challenges, such as the accumulation of per- and polyfluoroalkyl substances (PFAS) and micro- and nanoplastics (MNPs) in living organisms. These pollutants, which are increasingly pervasive in the environment, pose significant health risks, including metabolic disruption, carcinogenesis, and neuroinflammation. Recognizing the urgency of this issue, institutions such as the US Department of Health and Human Services have initiated research programs to develop methods to monitor and remove these contaminants from the human body. Here, our preliminary findings suggest that therapeutic apheresis may represent a promising approach for reducing environmental toxins, alongside its established role in lowering lipoproteins and inflammatory biomarkers, and warrants further systematic clinical investigation. This is based on the hypothesis that PFAS and MNPs form aggregates with lipids and proteins, suggesting that therapeutic apheresis could be an ideal method for clearance. In the initial results, we observed decreases in both PFAS and MNP levels in patient blood and sequestered plasma following double filtration plasmapheresis, although responses varied across individuals and analytes. Tissue and in vitro studies using imaging and functional bioassays may be suitable for demonstrating the potential of apheresis to remove PFAS and MNPs, as well as for assessing the impact of MNPs on cell viability, inflammation, and secretome profiles. These findings could serve as clinical surrogates to guide treatment frequency and evaluate the sustainability of therapeutic apheresis in mitigating health risks associated with environmental pollutants. While larger, well-designed clinical trials are needed to confirm these benefits, these early results could lay the groundwork for future research and clinical applications.

## Introduction

Therapeutic apheresis has undergone remarkable evolution since its inception about four decades ago, progressing from the early days of therapeutic plasma exchange (TPE) and double filtration plasmapheresis to encompass a broad spectrum of extracorporeal therapies. Our center is the world’s largest institution dedicated to therapeutic apheresis, performing up to 10,000 treatments annually across modalities such as lipoprotein apheresis, immunoadsorption, TPE, extracorporeal photopheresis, and erythrocyte apheresis. Over the past 25 years, we have established a unique longitudinal biobank comprising more than 8000 patient samples and biopsies, providing an unparalleled resource for translational research and clinical innovation.

Initially developed to manage severe dyslipidemias and cardiovascular diseases (1, 2), therapeutic apheresis has since expanded its clinical reach. It is now recognized as a valuable intervention for neurodegenerative disorders, chronic inflammation, autoimmune diseases, post-infectious syndromes, and age-related pathologies (3–6). This expansion reflects a growing appreciation of the central role that lipids and lipid-associated molecules play not only in cardiovascular health but also in neurodegeneration, immune dysregulation, and systemic aging.

In recent years, the global medical community has faced an unprecedented environmental health crisis: the pervasive contamination of human populations with per- and polyfluoroalkyl substances (PFAS) and micro- and nanoplastics (MNPs). More than 4700 PFAS compounds have been identified in commercial use and the environment, with biomonitoring studies revealing that over 99% of the US population has detectable PFAS in the blood. Approximately 200 million Americans have been exposed to PFAS-contaminated drinking water, and these compounds exhibit biological half-lives ranging from 4.8 to 8.5 years, persisting in the human body for decades (7). PFAS exposure is robustly linked to elevated LDL-cholesterol (LDL-C), metabolic syndrome, kidney and testicular cancer, and endocrine disruption (8–11). Simultaneously, MNPs have emerged as ubiquitous contaminants: individuals ingest an estimated 39,000–52,000 MNP particles annually, with total exposure rising to 74,000–121,000 particles per year when inhalation is included (12). MNPs have been detected in human blood, placenta, lung tissue, and even the brain, and inhalation of approximately 2.1 μg of airborne plastic particles per day is associated with increased risks of cardiopulmonary and lung cancer mortality (13, 14). In recognition of the scale and urgency of this emerging environmental health challenge, the Advanced Research Projects Agency for Health (ARPA-H), an agency within the US Department of Health and Human Services (HHS), announced STOMP: Systematic Targeting Of MicroPlastics, a $144 million program to create the definitive toolbox for measuring, researching, and affordably removing MNPs in the human body. However, prevention and environmental remediation alone are insufficient (15); there is a critical need for in vivo diagnostic tools and effective strategies to clear these contaminants from the human body.

In this study, we present the first data on therapeutic apheresis as a novel approach to removing PFAS and microplastics from the human body, along with initial evidence supporting a comprehensive strategy for in vivo diagnostic and functional monitoring. Our findings could lay the foundation for a new paradigm in the management of environmental toxin exposure and its associated disease burden.

## Results and discussion

### Targeting lipoproteins and inflammatory markers through extracorporeal therapeutic apheresis

Therapeutic apheresis is a well-established treatment modality for managing lipid-related cardiometabolic and neurometabolic diseases, effectively reducing circulating lipid levels and associated pro-inflammatory and pro-atherogenic factors, thereby mitigating disease progression (2, 16–18). Here, we confirm this by showing that the systemic inflammatory marker C-reactive protein (CRP) and fibrinogen were significantly reduced after each of two double-filtration plasmapheresis (DFPP) sessions (Figure 1, A and B). Furthermore, the lipoproteins LDL-C and Lp(a) were significantly reduced after each DFPP session, with no further reduction in post-treatment values between the first and second sessions (Figure 1, C and D).

MNPs are ubiquitous in the environment (19) and have been increasingly recognized as significant disruptors of endocrine, metabolic, cardiovascular, and neurological functions, as demonstrated by our work and that of others (15, 20–24). Mechanistically, PFAS, microplastics, and lipids act synergistically to sustain chronic inflammation and accelerate cellular aging. Both PFAS and MNPs are independently and jointly correlated with elevated LDL-C and increased risk of cardiometabolic diseases (25).

Biophysical studies demonstrate that PFAS partition to the surfaces of LDL and VLDL particles, stabilizing these lipoproteins and elevating circulating cholesterol levels (26). Furthermore, PFAS and MNPs can form macromolecular aggregates, so-called “corona molecules,” with lipoproteins, facilitating their systemic persistence and biological activity (27, 28). The formation of these coronas not only influences the environmental fate and transport of MNPs but also affects their biological interactions, including cellular uptake, membrane integrity, and potential toxicity (29, 30). Moreover, the specific composition of the corona, whether dominated by lipids, proteins, or other environmental factors, can either mitigate or exacerbate the toxicological effects of MNPs (31–33). Notably, PFAS molecules adsorb onto MNP surfaces at rates up to 250 times higher on environmentally aged plastics, forming combined molecular complexes whose toxicity exceeds that of either compound in isolation, thereby disrupting lipid metabolism, neurogenesis, and inflammatory signaling (33, 34). These findings underscore the complexity of the toxic burden now facing human populations: PFAS and MNPs circulate not as isolated molecules, but as heterogeneous, lipid-protein-toxin aggregates that evade conventional pharmacological interventions.

Given this complexity, pharmacological strategies are fundamentally unsuited to the comprehensive removal of such aggregates from the human body. Building on previous lipidomic and proteomic investigations (2, 5, 35, 36), we note that therapeutic apheresis is a clinically established extracorporeal method capable of removing, at scale, lipoproteins and inflammatory proteins and may therefore be well-suited to investigating whether lipid- or protein-associated environmental contaminants can also be reduced. Using two different apheresis systems based on filtration or adsorption, we confirmed by Raman spectroscopy that cholesterol levels decrease after apheresis, as indicated by characteristic Raman bands at specific wavenumbers. Cholesterol exhibits distinct and reproducible spectral features that enable reliable detection, and both filter systems showed similar reductions in the peak corresponding to cholesterol at wavenumbers between 1440 and 1465 cm−1 (Figure 2). Similar reductions were observed at wavenumbers around 1155 cm−1, which are also associated with lipids and fatty acids. This signal also decreased after apheresis with both methods studied (data not shown).

Raman spectroscopy is well-suited for microplastic identification because of strong polymer-specific fingerprints. MNPs, such as polystyrene (PS) and the polyamides 6 and 66, display characteristic Raman signatures in spectral regions comparable to those of cholesterol. However, in complex matrices such as plasma, performance is limited by spectral interference, fluorescence background, and analytical throughput. Sensitivity further decreases for particles below ~1 μm, where insufficient polymer mass within the laser focal volume may preclude a signal distinguishable from noise. In contrast, cholesterol is detected as a distributed molecular species, where the Raman signal arises from a large ensemble of molecules within the excitation volume rather than from discrete particles. Therefore, given the limited sensitivity and overlap of spectral features in plasma, any potential polymer-specific signals could not be reliably distinguished from background and lipid-associated contributions in this dataset. Accordingly, this method cannot be used to determine whether MNPs contribute to the observed Raman peaks before and after apheresis.

Here, other methods, such as advanced vibrational spectroscopy, mass spectrometry, particle separation and characterization, and multi-omics profiling, can be employed to overcome the limitations of Raman spectroscopy in complex plasma matrices and to detect and characterize corona aggregates comprising lipoproteins, MNPs, and PFAS.

### Targeting PFAS through extracorporeal therapeutic apheresis

To determine whether extracorporeal apheresis can reduce the levels of PFAS in patient plasma, we employed two independent laboratories to quantify PFAS in a small cohort of individuals using liquid chromatography-tandem mass spectrometry (LC-MS/MS). At Medizinisches Labor Bremen, a certified German reference laboratory for environmental toxins, reductions of up to 25% in perfluorooctanoic acid (PFOA), perfluorooctane sulfonic acid (PFOS), perfluorononanoic acid (PFNA), and perfluorohexane sulfonic acid (PFHxS) were observed (Figure 3).

Similarly, at Creative Biostructure, PFAS levels in four individuals were measured, and reductions in multiple PFAS were observed after apheresis (Figure 4). In these individuals, the reductions were even more pronounced. On average, the following decreases were observed: PFOS, 48.9%; PFOA, 43.5%; PFNA, 55.0%; perfluorododecanoic acid (PFDoDA), 75.8%; and perfluorodecanoic acid (PFDA), 47.1%.

To determine whether aggregates, specifically, coronas composed of lipoproteins and PFAS or MNPs, are removed by apheresis, plasma samples collected before and after apheresis, as well as the eluate, should be subjected to separation using, for example, Asymmetric Flow Field-Flow Fractionation coupled with Multi-Angle Light Scattering (AF4-MALS). AF4-MALS separates particles and aggregates by hydrodynamic size, typically ranging from 10 nm to over 100 μm, under gentle, low-shear conditions that preserve native corona and aggregate structures (37). This technique is highly compatible with plasma and other complex biological matrices, enabling high-resolution fractionation of heterogeneous populations such as high-density lipoprotein (HDL, 7.5–14 nm), low-density lipoprotein (LDL, 19–29.5 nm), very low-density lipoprotein (VLDL, >30 nm), and nanoparticle or MNP aggregates (38). Importantly, AF4-MALS is widely used to isolate and characterize protein and lipoprotein coronas on nanoparticles in plasma, preserving their native composition and enabling detailed analysis of corona heterogeneity (39). The technique can be directly coupled to downstream mass spectrometry or FTIR, allowing comprehensive compositional profiling of separated fractions, including identification of proteins, lipids, and potential PFAS or MNP components.

### Targeting MNPs through extracorporeal therapeutic apheresis

MNP analysis relies on complementary analytical techniques, including vibrational spectroscopy (Fourier-transform infrared spectroscopy [FTIR] and Raman spectroscopy), pyrolysis gas chromatography-mass spectrometry (Py-GC-MS), and microscopy-based approaches such as scanning electron microscopy (SEM; 40–43). FTIR is a widely used non-destructive method suitable for particles typically > 10–20 μm, with micro-FTIR enabling analysis down to approximately 5 μm (41, 44). Raman spectroscopy provides higher spatial resolution and can detect particles as small as ~1 μm, making it particularly useful for smaller microplastics (45, 46). Py-GC-MS offers highly sensitive quantitative analysis, including nanometer-scale particles and fragments, but is destructive and does not preserve particle morphology (47–49). Microscopy-based techniques such as SEM enable detailed visualization of particle morphology, including submicron and nanoscale particles, but generally require complementary methods for chemical identification (50). As each method has distinct strengths and limitations, combined analytical approaches are often necessary for comprehensive MNP detection and characterization across different size ranges (41, 49).

To ensure reliable and comparable results, method validation, harmonization, and standardization are essential (51). However, widely validated and standardized methods remain lacking, and inconsistent data reporting between laboratories persists. Developing suitable reference materials that reflect real-world microplastics, encompassing diverse polymers, sizes, shapes, and aging states, is critical to advancing research. Projects such as EUROqCHARM are addressing these gaps by developing blueprints for standards, policy, and legislative recommendations.

Sampling and storage are highly susceptible to contamination because MNPs are ubiquitous in the environment and can be introduced into samples almost instantaneously. To maintain data integrity, researchers must avoid plasticware wherever possible and adhere to rigorous quality standards (52, 53). Positive and negative controls should be included at every stage to validate findings and ensure measurement accuracy.

Recently, we demonstrated using ATR-FTIR that extracorporeal DFPP may also remove MNPs from human blood (20). We have now confirmed these findings using a range of currently available methodologies to measure MNPs in plasma, both quantitatively and qualitatively, before and after apheresis.

First, we employed two independent laboratories to quantify MNPs in a small cohort of individuals using Py-GC-MS. At Creative Biostructure, in blood samples prepared with previous removal of organic material, polyethylene (PE) levels decreased in four patients, while polyvinyl chloride (PVC) levels decreased in three of four patients following apheresis. In contrast, polypropylene (PP) decreased in only one patient, increased in two, and was not detectable in the fourth patient. Polystyrene (PS) and polyamide 66 were detectable in only one of the four patients. Notably, PS increased after apheresis, whereas polyamide 66 decreased (Figure 5A). Overall, these findings suggest that therapeutic apheresis may reduce or redistribute circulating MNP levels. The varying effects among polymer types likely reflect the small cohort size, biological variability, and the current limitations of MNP analytical methods. Furthermore, the variability observed among different polymer types and between individuals suggests that circulating MNP concentrations may represent a dynamic equilibrium between blood and tissue compartments. Similar to other persistent environmental contaminants, an initial reduction in circulating MNPs following therapeutic apheresis may be partially offset by mobilization from tissue reservoirs. Consequently, repeated treatment sessions may be required before a sustained decrease in circulating MNP levels becomes apparent. Future longitudinal studies with larger cohorts and serial measurements will be needed to test this hypothesis.

Different studies suggest that neutrophil extracellular traps (NETs) may interact with a range of environmental particulates and nanoparticles, including components of air pollution and engineered nanomaterials, likely through electrostatic and protein-mediated binding within the NET scaffold (54, 55). We have previously observed increased NET markers following apheresis, consistent with neutrophil activation and/or redistribution during extracorporeal blood processing (56). For the first time under hospital-exempt exploratory use, we applied a novel adsorber (NucleoCapture, Santersus AG) during apheresis. This adsorber is designed to bind and remove extracellular nucleic acid structures, including NET-derived components, in conjunction with apheresis technology. At Measurlabs, Py-GC-MS was performed following extensive sample preparation to minimize lipid interference. In the two exploratory cases, patients underwent combined DFPP and NucleoCapture treatment rather than DFPP alone. In one patient, complete removal of all detectable PS and PP was observed, while in the second patient, complete removal of PE, PP, and polyethylene terephthalate (PET) was achieved. No other MNP species were detectable in either patient (Figure 5, B and C). Although the sample size is insufficient for definitive conclusions, the findings represent the greatest reduction in measured MNP burden in the present study and raise the possibility that selective adsorption of extracellular nucleic acid-containing structures may enhance the clearance of circulating MNPs beyond filtration alone. One potential explanation is that a proportion of circulating MNPs may be associated with extracellular chromatin, NETs, extracellular vesicles, or other macromolecular carrier complexes, which could be co-removed by adsorption-based technologies such as NucleoCapture. However, this remains speculative and requires direct experimental validation. If such associations are confirmed, they could provide a mechanistic basis for extracorporeal removal strategies targeting circulating environmental particles through therapeutic apheresis approaches employing selective filters and adsorbers. It should be noted that both patients had pulmonary diseases associated with smoking and other contributing factors, although the relevance of these clinical characteristics to MNP clearance remains uncertain.

To estimate the number of smaller particles in human plasma before and after apheresis, we employed the Germans Trias i Pujol Research Institute (IGTP), which uses Nile red staining of pre-digested samples to remove organic matter. Stained particles are then detected by flow cytometry (57). Analysis of four patient samples showed an average reduction of ~70% after apheresis (Figure 6). Notably, this method provides particle counts but does not definitively determine particle identity, that is, whether they are truly microplastics, since Nile red stains all hydrophobic substances, though limited by factors like polymer crystallinity, steric hindrance, and fluorescence quenching.

These results in small cohorts suggest that DFPP indeed has the potential to reduce the number of MNPs in human blood. It should be noted that these observations reflect acute post-procedural changes in circulating PFAS and MNP levels and cannot be directly extrapolated to long-term changes in total body burden. Whether repeated apheresis sessions can modify steady-state levels remains unknown and requires longitudinal investigation. In addition to the direct removal of these particles, it should be noted that apheresis exerts several other effects, including reductions in lipoproteins, inflammatory markers, and heavy metals (2, 5, 17, 18, 36, 58). Consequently, the overall pro-inflammatory milieu in the body may be reduced after apheresis, which could, in turn, mitigate the adverse effects of MNPs, whether acting alone or in aggregates with, for example, lipids.

### In vitro monitoring of MNPs

Functional assays are essential for systematically evaluating the biological effects of MNPs on cellular function. They provide critical insights into how MNPs interact with biological systems, including their effects on cell death, metabolism, structural integrity, and signaling pathways. The need for functional assays stems from the complexity of MNP-induced effects, which often involve multiple cellular and molecular mechanisms. These assays should utilize a variety of cell types, including brain cells, adrenal cells, islets, endothelial cells, liver cells, and semen, to comprehensively assess the impact of MNPs on cellular function. By measuring key parameters such as cytokine levels, hormone secretion, lipid profiles, gene expression, and cellular morphology, functional assays can provide a multidimensional understanding of MNP-induced changes. For example, cytokine profiling can reveal inflammatory responses, while lipid analysis can shed light on disruptions in cellular membranes or metabolic pathways.

A critical aspect of both the quantitative measurements and the functional assays is the inclusion of appropriate positive and negative controls. In this study, an important advantage is the availability of plasma samples from the same patients before and after treatment. This paired design minimizes confounding from potential contaminants originating from plastic materials used during sampling or analysis, as sample collection and processing were performed identically across conditions; thus, observed differences are likely to reflect true biological effects. This strengthens the reliability of the results and enables monitoring of treatment efficacy.

In the context of therapeutic apheresis, functional assays can further be applied to assess reductions in MNP-induced cellular effects following treatment, potentially supported by machine learning approaches. Correlating these findings with clinical outcomes may help validate therapeutic efficacy and refine treatment protocols.

In summary, functional assays are a cornerstone of MNP research, providing the framework for evaluating the biological impact of MNPs, validating diagnostic methods, and optimizing therapeutic strategies.

Recently, we identified PS in human eyelid skin using Raman spectroscopy (Figure 7, A–C), demonstrating our ability to detect MNPs in human tissue. We are currently evaluating in vitro systems, including human sperm motility assays and endothelial cell models, to assess fertility- and cardiovascular-related effects associated with MNP exposure and to investigate the impact of apheresis. Preliminary results using the human adrenal cell line NCI-H295R indicated a trend towards PS-induced cell death, as reflected by increased lactate dehydrogenase (LDH) release. Incubation of PS particles with patient serum collected before apheresis did not alter this LDH release compared to PS alone. In contrast, incubation with serum collected after apheresis showed a trend towards reduced LDH release, indicating that apheresis induces or releases inhibitory factors in patient serum that attenuate microplastic-induced cytotoxicity in human adrenal cells (Figure 7D).

These assays confirm the presence of MNPs across a range of tissues, exemplified here by human eyelid skin. Furthermore, they highlight the complexity of assessing the effects of MNPs within complex biological matrices such as serum/plasma, as well as in the human body as a whole.

### Conclusion

This study highlights the potential of therapeutic apheresis as a novel approach to addressing the growing health burden posed by environmental pollutants, including PFAS and MNPs. By leveraging advanced analytical techniques such as LC-MS/MS, Py-GC-MS, and flow cytometry, we observed reductions in circulating levels of these contaminants, in addition to its established efficacy in lowering lipoproteins and inflammatory markers. These findings suggest that apheresis may reduce selected circulating PFAS and MNP-associated signals and may mitigate their systemic effects, including endocrine disruption, cardiovascular risks, accelerated aging, and cellular degeneration, by disrupting lipid-protein-toxin aggregates (coronas) and reducing the pro-inflammatory milieu (Figure 8).

Compared with conventional TPE, therapeutic apheresis using DFPP and/or absorption systems may offer additional advantages for removal of environmental toxicants. While TPE can also remove circulating toxic substances, it relies on replacement with donor plasma or albumin, which may itself introduce new contaminants, including environmental toxins, inflammatory mediators, or other unwanted biological components from the donors. In contrast, DFPP and/or adsorption directly remove targeted substances from the patient’s plasma without donor replacement, thereby minimizing the risk of secondary exposure and enabling more selective and potentially personalized detoxification strategies. A possible mechanism underlying the observed reduction in circulating MNPs is the physical retention of larger particles by membrane filters whose pore sizes prevent their passage. In adsorption-based systems, MNPs may additionally bind to the adsorber material either directly or indirectly through interactions with plasma proteins, lipoproteins, or immune complexes. Smaller particles may pass through the filtration membranes and accumulate in the eluate.

Extracorporeal therapeutic apheresis is generally considered a safe and well-tolerated procedure when performed in experienced centers under standardized protocols. Reported adverse events are usually mild and transient, while serious adverse events are rare. The most commonly reported side effects in the literature include mild hypotension, dizziness, fatigue, nausea, and transient citrate-related symptoms such as paresthesia or muscle cramps due to anticoagulation-induced hypocalcemia (59). Allergic reactions may occur particularly in procedures involving plasma replacement (60, 61); however, these reactions are uncommon and are less likely in DFPP and adsorption-based systems that avoid donor plasma administration. Hypertensive episodes have also been described occasionally, although significant blood pressure alterations are generally rare and usually manageable with routine monitoring during treatment.

Limitations of this study include the small sample size, heterogeneity of patient indications and apheresis systems, the exploratory nature of the MNP assays, the risk of environmental contamination during sampling and processing, and the absence of long-term clinical outcome data. Furthermore, while the observed changes across lipid, PFAS, and MNP measurements are consistent with extracorporeal reduction processes, the lack of consistent individual-level pairing across datasets limits conclusions regarding a shared or unified clearance mechanism.

Figure 9 conceptualizes the mechanisms of MNP toxicity, illustrating how MNPs and PFAS may interact with biological systems by forming aggregates and corona complexes that can amplify systemic toxicity and exacerbate adverse biological effects. The “Sorcerer’s Apprentice” paradigm illustrates the amplification of toxic effects through aggregation. The “Snowball” paradigm depicts how MNPs serve as carriers of lipids, viruses, bacteria, and environmental toxins, while the “Trojan Horse” paradigm highlights cellular uptake and systemic transport. These mechanisms underscore the urgent need for therapeutic interventions, such as apheresis, to disrupt these harmful processes and reduce the health risks associated with environmental pollutants.

While these findings are encouraging, they represent only the initial steps towards understanding the therapeutic potential of apheresis. As mentioned above, integrating functional assays into future research is critical for systematically evaluating the biological effects of MNPs and the efficacy of their removal. These assays will enable researchers to assess cellular responses, including inflammation, metabolic changes, and structural damage, providing a deeper understanding of how MNPs contribute to disease progression.

Measuring and characterizing MNPs pose a significant analytical challenge, as no single technique can currently address the wide range of particle sizes, shapes, polymer types, and environmental conditions in which they occur. Each analytical method has specific strengths and limitations, necessitating a multi-technique approach to obtain comprehensive and reliable data. Additionally, the quality of published research in this field varies significantly, underscoring the need for technique refinement, proper controls, and standardization to ensure the credibility of results (62).

Furthermore, long-term clinical studies with larger cohorts are essential to establish a clear correlation between diagnostic results and clinical outcomes. These studies should also explore the frequency and duration of apheresis treatments required to achieve sustained health benefits. Monitoring treatment success using functional assays and follow-up studies will be vital for optimizing therapeutic protocols and ensuring their long-term effectiveness.

The preliminary observations obtained with combined DFPP and selective nucleic acid adsorption further suggest that increasingly targeted extracorporeal approaches may enhance removal of environmental toxicants and their associated macromolecular carrier complexes. Future studies should determine whether NETs, extracellular chromatin, extracellular vesicles, and protein coronas contribute to the systemic transport of MNPs and PFAS and whether selective adsorption technologies can improve clearance beyond filtration alone.

In conclusion, this work provides an important foundation for addressing the growing challenge posed by environmental factors to human health. By integrating advanced analytical diagnostics, therapeutic apheresis, and functional bioassays, it may become possible to develop comprehensive strategies to reduce the biological burden and health risks associated with PFAS and MNP exposure. These findings further support a broader therapeutic concept in which apheresis is combined with increasingly selective filtration and adsorber technologies, enabling personalized extracorporeal treatment strategies tailored to specific pathological burdens, including cardiovascular diseases, chronic inflammation, autoimmune disorders, neurovascular diseases, and environmental toxic exposures. Such an approach could support the development of therapeutic apheresis as a precision-medicine platform for selected pathological burdens, provided that analytical validity, clinical efficacy, and long-term safety are confirmed in prospective studies.

## Materials and methods

### Patient cohorts and clinical parameters

Blood and tissue samples were collected immediately before and after 1–2 apheresis procedures. The target population comprised adult patients scheduled to receive clinically indicated therapeutic apheresis for conditions for which apheresis is recommended according to the guidelines of the American Society for Apheresis (ASFA), including lipid disorders (e.g., familial hypercholesterolemia or elevated Lp(a)) and autoimmune diseases (63). All participants were informed about the study and provided written informed consent. Samples and data were analyzed in a pseudonymized manner in accordance with the requirements of the institutional ethics committee. Monitoring serum/plasma and eluate samples before and after apheresis is part of the routine and quality control in our center. LDL-C and Lp(a) levels were measured in patients’ blood samples using a Roche automated clinical chemistry analyzer, MODULAR (Roche Diagnostics GmbH, Mannheim, Germany), as described previously (6). Fibrinogen and C-reactive protein (CRP) were measured using the Cobas-System (Roche Diagnostics AG, Basel, Switzerland).

### Therapeutic apheresis

DFPP procedures were performed using the Inuspheresis System (Ayus Medical Devices AG, Steinhausen, Switzerland). Other lipoprotein apheresis procedures were performed using the Kaneka MA-03 apheresis system with the LIPIDsmart^™^ adsorber (Kaneka Corporation, Osaka, Japan). In two exploratory cases, DFPP was combined with a selective extracellular nucleic acid adsorption device (NucleoCapture, Santersus AG, Zurich, Switzerland). Following plasma separation and fractionation, plasma was passed through the NucleoCapture adsorber before reinfusion. NucleoCapture is designed to bind extracellular nucleic acid-containing structures, including cell-free DNA, nucleosomes, and NET-derived chromatin complexes. These treatments were performed under hospital-exempt exploratory use and were analyzed separately from DFPP-only procedures.

### MNP measurements

Raman spectroscopy was used to quantify MNPs and cholesterol in blood samples obtained before and after apheresis as previously described (64, 65).

Furthermore, MNP levels were quantified in plasma samples obtained before and after apheresis by Py-GC-MS in two independent laboratories (Measurlabs [Helsinki, Finland] and Creative Biostructure [Shirley, NY, USA]) using validated protocols.

Nanoparticles were quantified by Nile red staining followed by flow cytometry in blood samples obtained before and after apheresis by the Germans Trias i Pujol Research Institute (IGTP, Badalona, Spain) as described previously (57).

In formalin-fixed paraffin-embedded (FFPE) human eyelid tissue obtained from the Tumor and Normal Tissue Bank (TNTB), part of BioBank Dresden, Institute of Pathology, University Hospital Carl Gustav Carus, Technische Universität Dresden, Dresden, Germany, Raman spectroscopy and imaging were used for the measurement of PS particles using the alpha 300R Raman imaging system (WITec) with a 532 nm laser (laser power: 5 mW, objective: 100x).

### PFAS measurements

PFAS levels were quantified in blood samples obtained before and after apheresis, as well as in the corresponding eluates, by liquid chromatography-tandem mass spectrometry (LC-MS/MS) in two independent laboratories (Medizinisches Labor Bremen [Bremen, Germany] and Creative Biostructure) using validated certified protocols.

### Cell culture and cell death determination

NCI-H295R cells were cultured in DMEM/F12 supplemented with 2.5% Nu-Serum and 1% penicillin-streptomycin at 37°C in a humidified atmosphere containing 5% CO_2_. Cells were treated for 24 h with carboxylate-modified polystyrene latex beads (mean particle diameter 30 nm; Sigma-Aldrich, Merck KGaA, Darmstadt, Germany, L5155) at 100 μg/mL, with 2% serum obtained from patients before and after apheresis, or with a combination of both. After incubation, cell necrosis was assessed using a lactate dehydrogenase (LDH) cytotoxicity assay kit according to the manufacturer’s protocol (Promega, Madison, WI, USA, G1780).

## Figures and Tables

**Figure 1.**
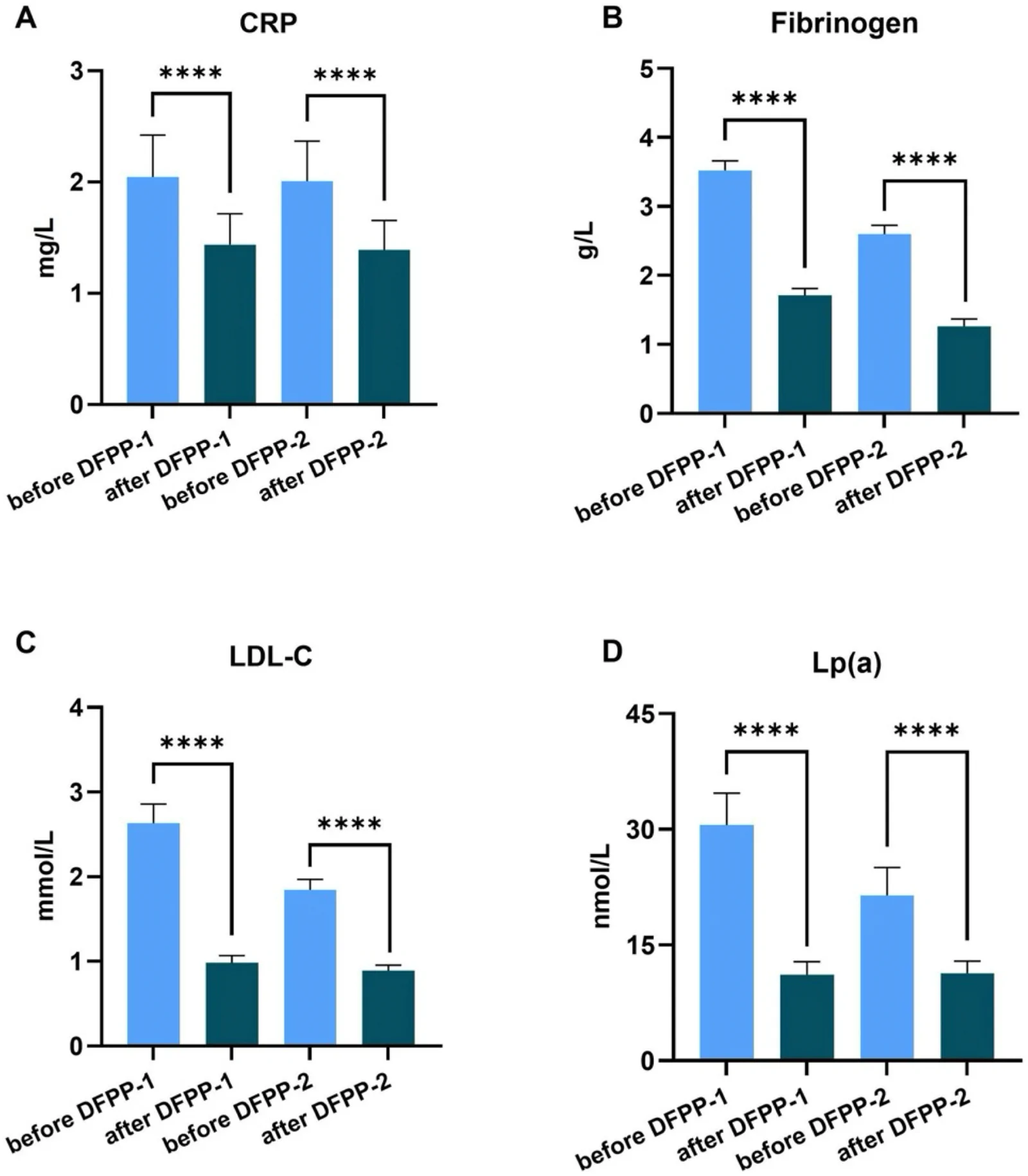
Reduction of CRP (A), fibrinogen (B), LDL-C (C), and Lp(a) (D) by DFPP treatment. In 34 individuals, the concentrations of the respective parameters were measured immediately before and after each DFPP session. Data for each group are presented as mean ± SEM. Student’s *t*-test, statistically significant differences between pre- and post-treatment values are indicated as **** *P* ≤ 0.0001.

**Figure 2.**
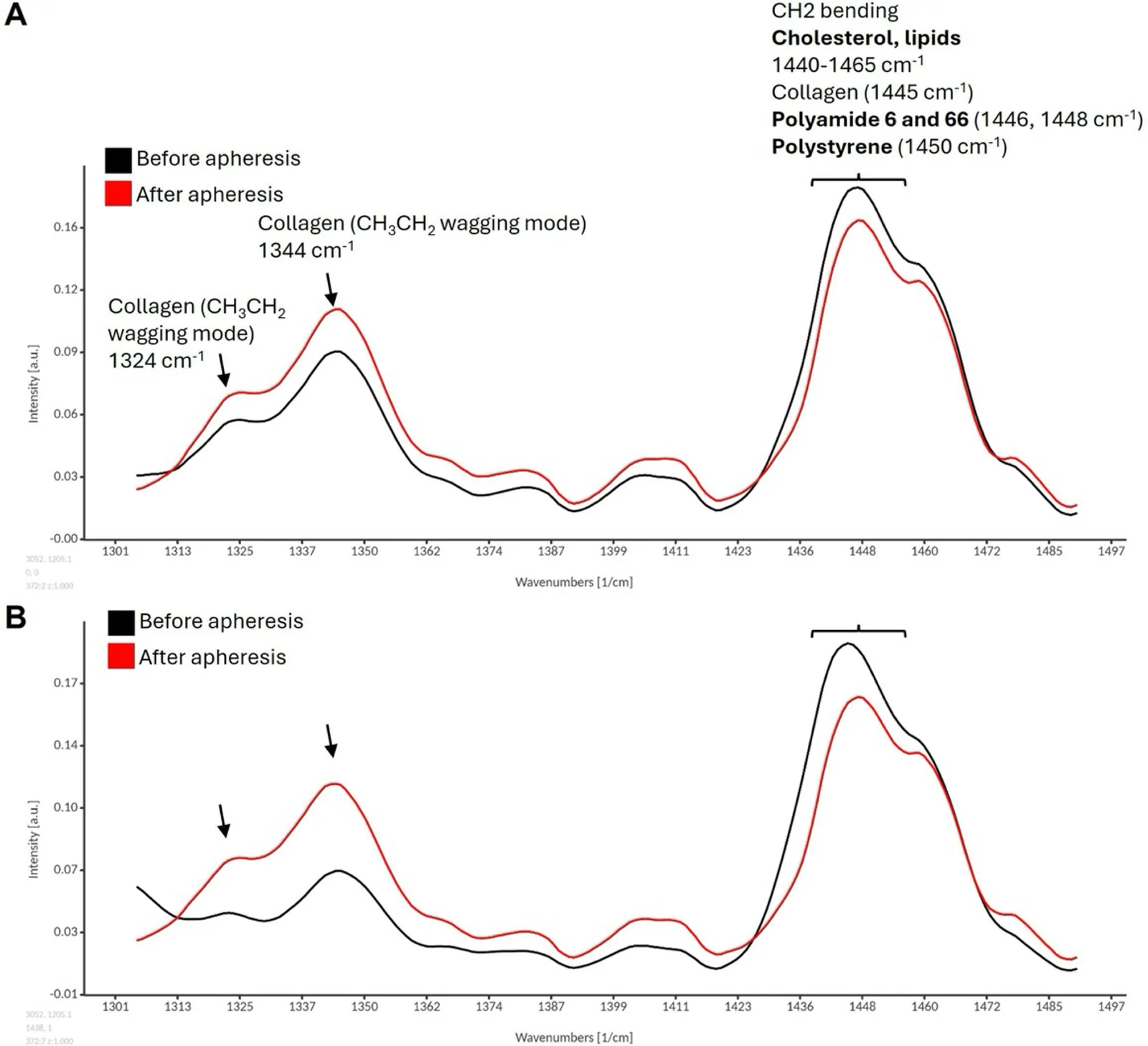
Reduction of cholesterol after apheresis. Using Raman spectroscopy, the levels of cholesterol and associated lipids in patient blood were measured before and after apheresis using two different apheresis systems, such as filtration and adsorption. (A) Inuspheresis. (B) LIPIDsmart^™^. The Raman peaks characteristic of cholesterol were attenuated after treatment.

**Figure 3.**
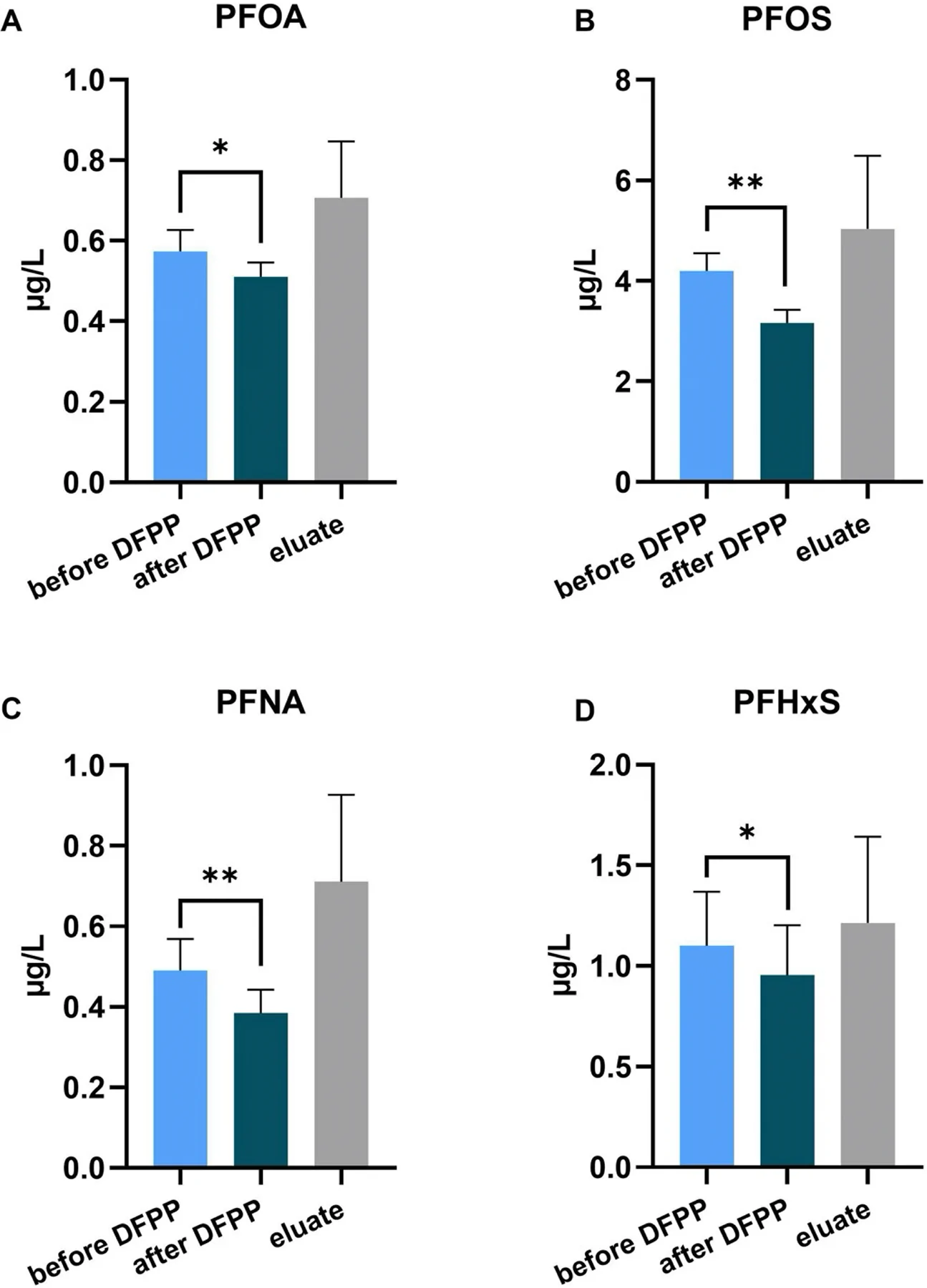
Reduction of different PFAS by DFPP treatment. Mean concentrations of PFOA (A), PFOS (B), PFNA (C), and PFHxS (D) in patients’ plasma before and after DFPP treatment, as well as in the corresponding eluates, were measured by LC-MS/MS. Data are presented as mean ± SEM from 14 individuals; Student’s *t*-test. Statistically significant differences between pre- and post-treatment values are indicated as * *P* ≤ 0.05 or ** *P* ≤ 0.01.

**Figure 4.**
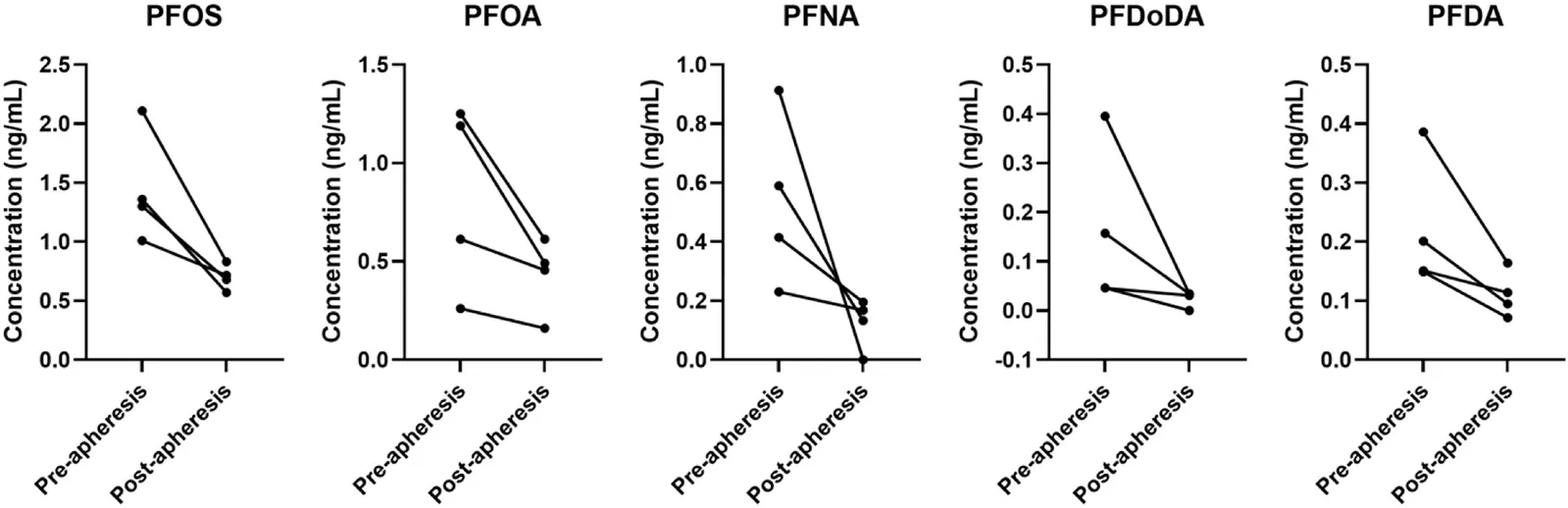
Individual-patient reductions in PFAS after a single DFPP session. Four patients underwent a single DFPP session. Blood samples collected before and after treatment were analyzed for PFAS by LC-MS/MS (Creative Biostructure). Each line in the diagrams corresponds to an individual patient.

**Figure 5.**
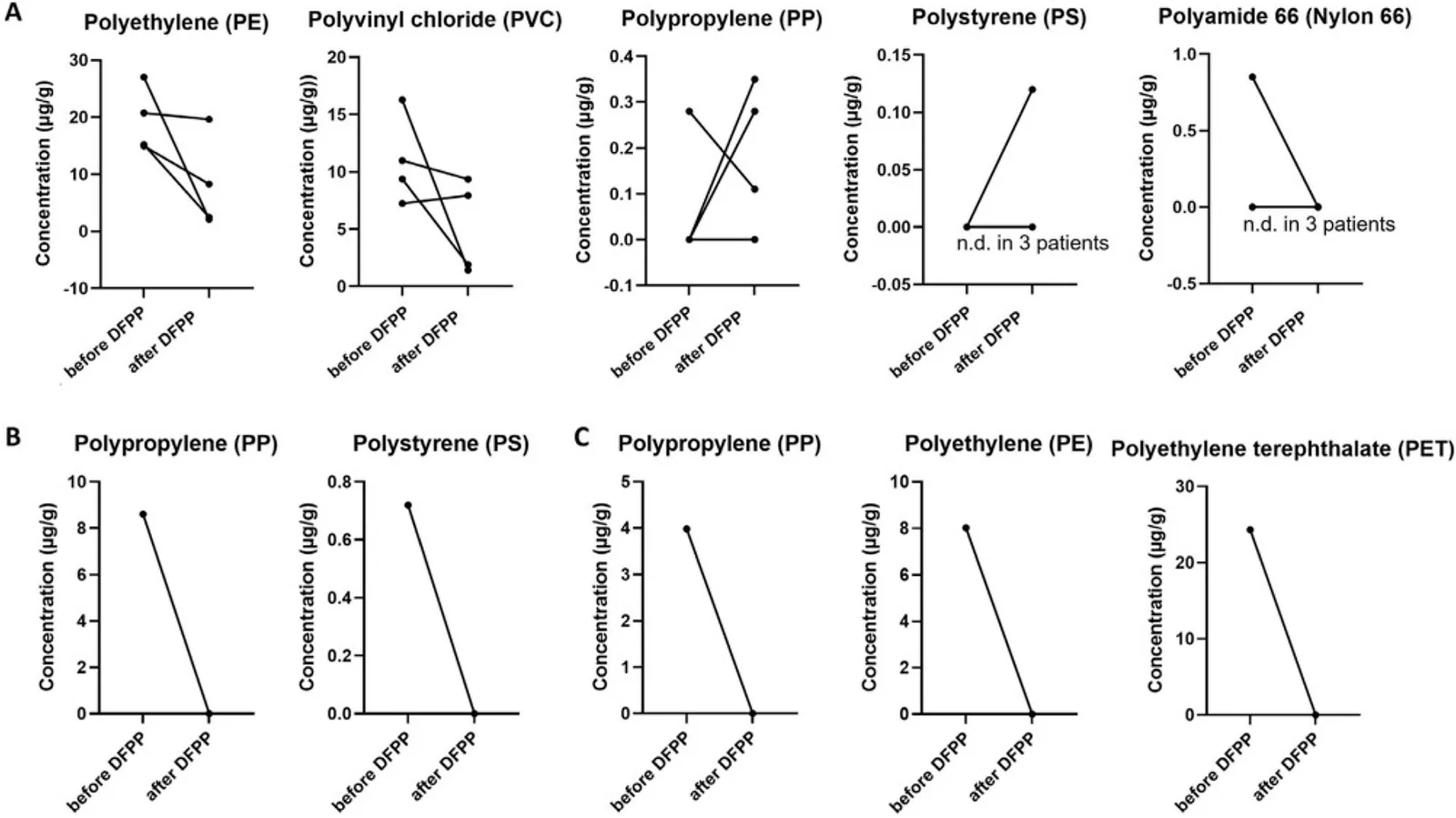
MNP measurements of patient plasma before and after DFPP treatment. (A) Four patients each underwent a single DFPP session. Blood samples collected before and after treatment were analyzed for MNPs using Py-GC-MS (Creative Biostructure). n.d., not detectable. Each line in the graphs corresponds to a single patient; for PS and polyamide, the line at the axis baseline corresponds to the three patients in whom the polymer was not detectable. (B + C) Two patients underwent a single combined DFPP + NucleoCapture session. Blood samples collected before and after treatment were analyzed for MNPs using Py-GC-MS (Measurlabs). Complete removal of several detected MNP species was observed following treatment.

**Figure 6.**
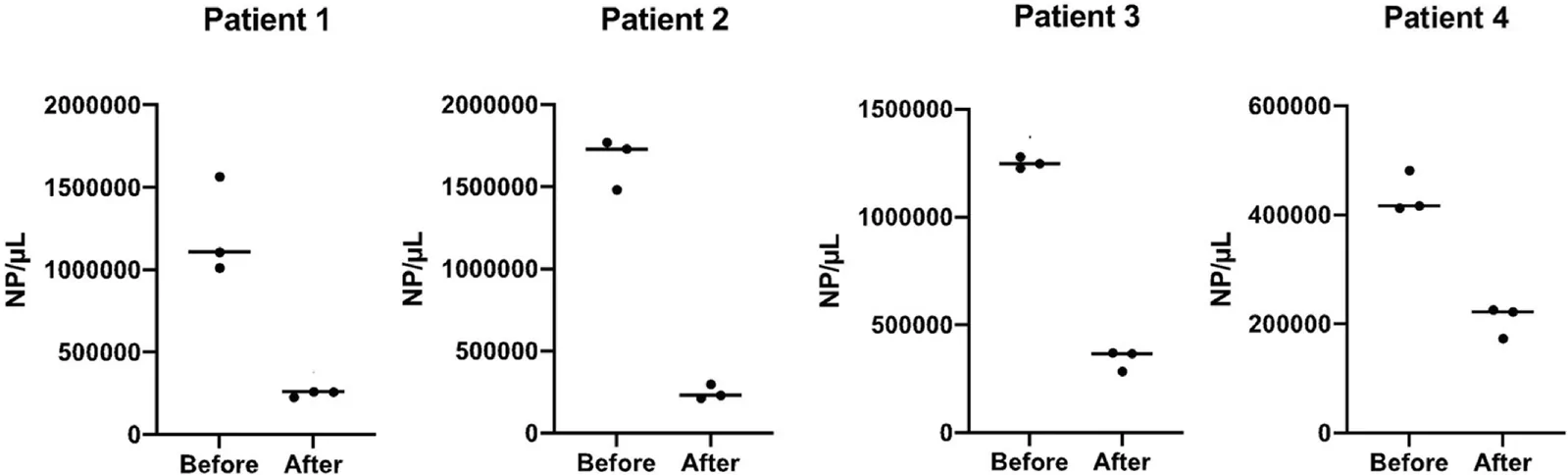
Measurement of nanoparticles in patient plasma before and after DFPP treatment. Four patients each underwent a single DFPP session. Organic material was digested from blood samples collected before and after the session, and samples were then stained with Nile Red dye. The stained particles were subsequently quantified by flow cytometry (IGTP). Statistical analysis was performed using a one-tailed Wilcoxon matched-pairs test.

**Figure 7.**
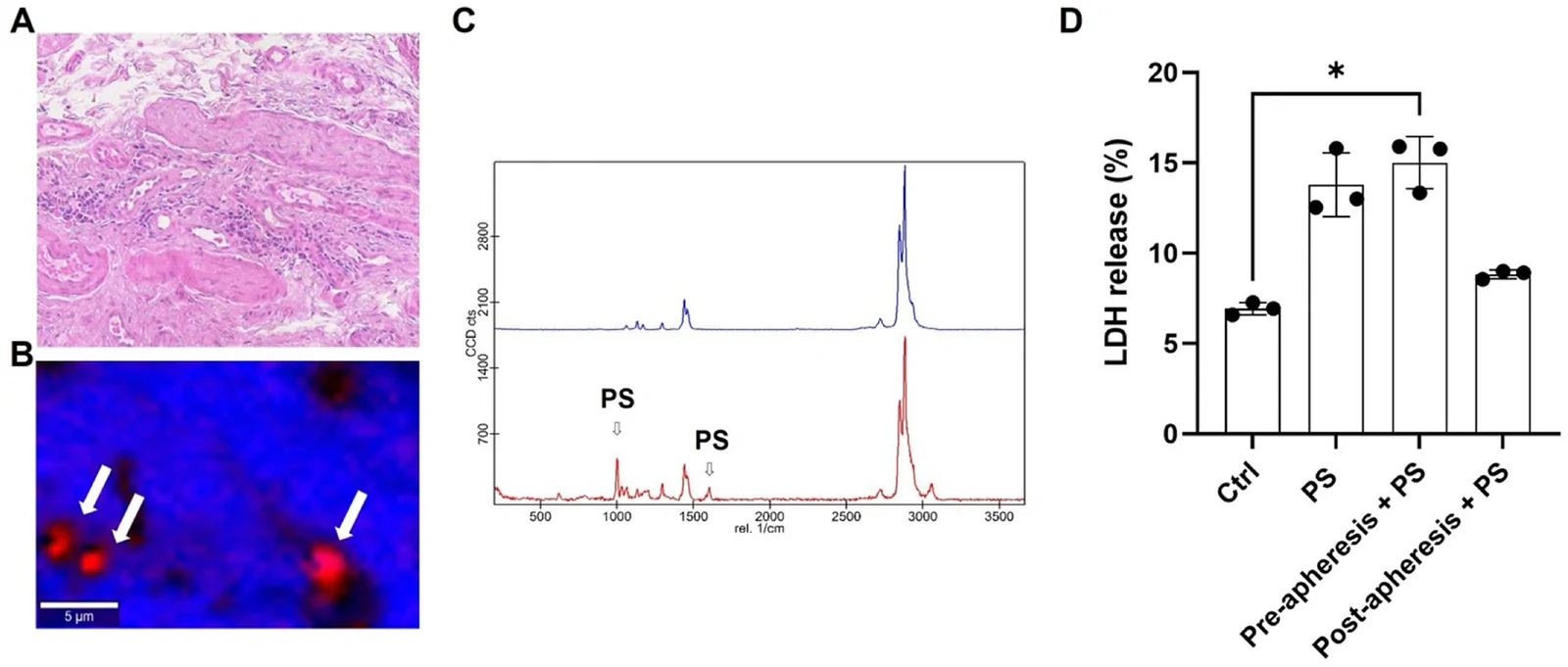
Microplastic detection in human tissue and effect in adrenal cells. A representative human eyelid skin specimen is shown in H&E staining (A), and Raman spectroscopy detects and spatially maps polystyrene (PS) particles indicated by arrows (B), confirming our ability to localize micro- and nanoplastics (MNPs) in human samples. Scale bar, 5 μm. (C) Top panel shows embedding media and tissue without MNPs. Bottom panel shows additional peaks corresponding to PS indicated by arrows. (D) The human adrenal cell line NCI-H295R was exposed to 30 nm PS particles (100 μg/mL), and cell death was assessed by measuring lactate dehydrogenase (LDH) release. In addition, NCI-H295R cells were exposed to PS particles incubated with serum obtained before or after apheresis treatment. Data are presented as means ± SEM. Statistical analysis was performed using the Kruskal-Wallis test followed by Dunn’s test. *P ≤ 0.05; *n* = 3. Ctrl, control; PS, polystyrene.

**Figure 8.**
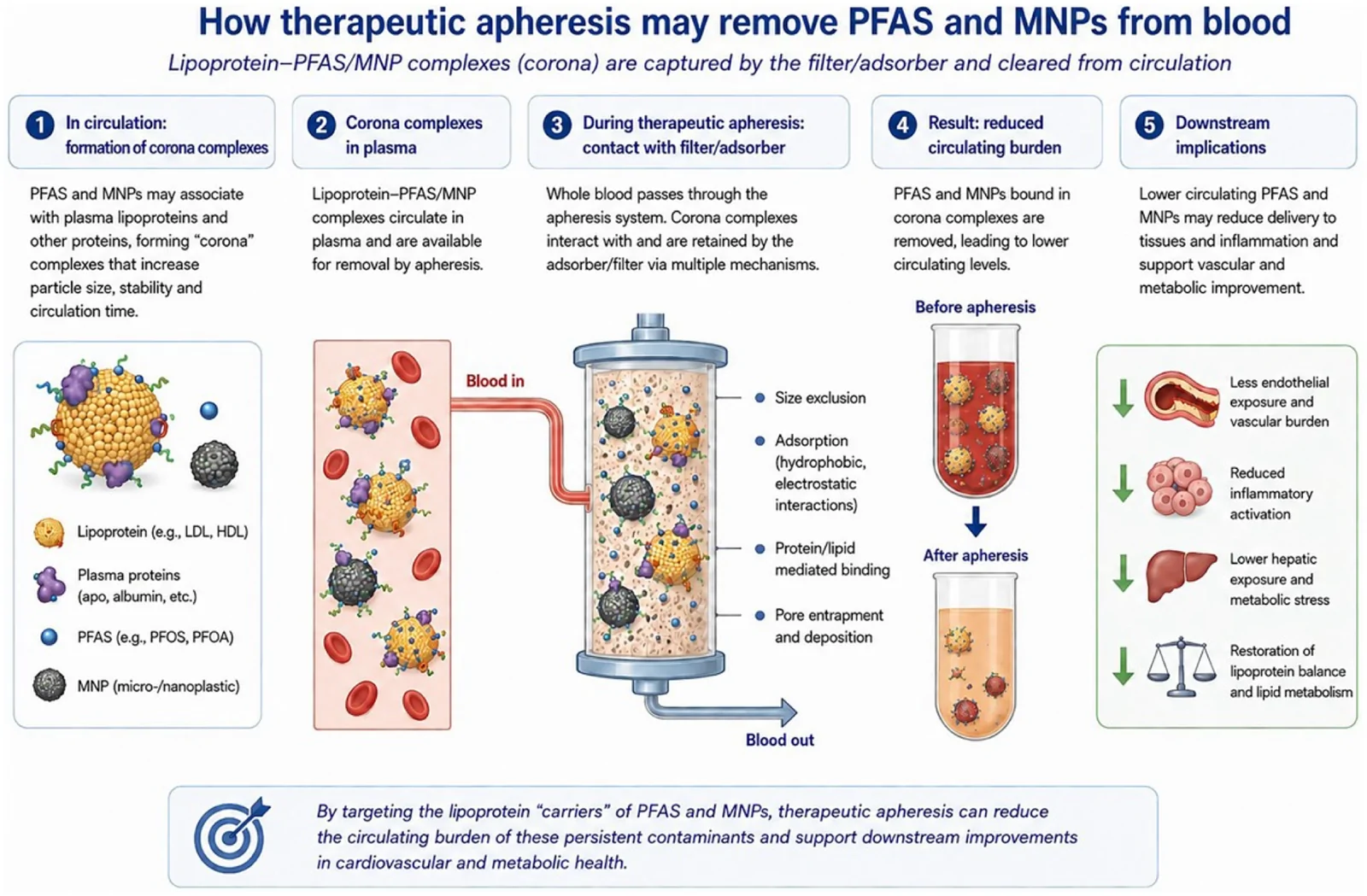
Schematic overview of how therapeutic apheresis may reduce circulating PFAS and MNPs by capturing lipoprotein–PFAS/MNP “corona” complexes. The workflow illustrates five stages: 1) Formation of corona complexes: PFAS and MNPs associate with plasma lipoproteins and proteins, forming larger, more stable circulating complexes; 2) Circulation of corona complexes: these lipoprotein–PFAS/MNP complexes remain in plasma and become accessible for removal by apheresis; 3) Apheresis interaction: as whole blood passes through the apheresis filter/adsorber, corona complexes are retained through size exclusion, adsorption, protein/lipid-mediated binding, and pore entrapment; 4) Reduced circulating burden: removal of these complexes decreases blood levels of PFAS and MNPs; 5) Downstream implications: lower systemic exposure supports reduced vascular and inflammatory burden, decreased hepatic stress, and improved lipoprotein and metabolic balance. ChatGPT (OpenAI) was used to generate the schematic figure.

**Figure 9.**
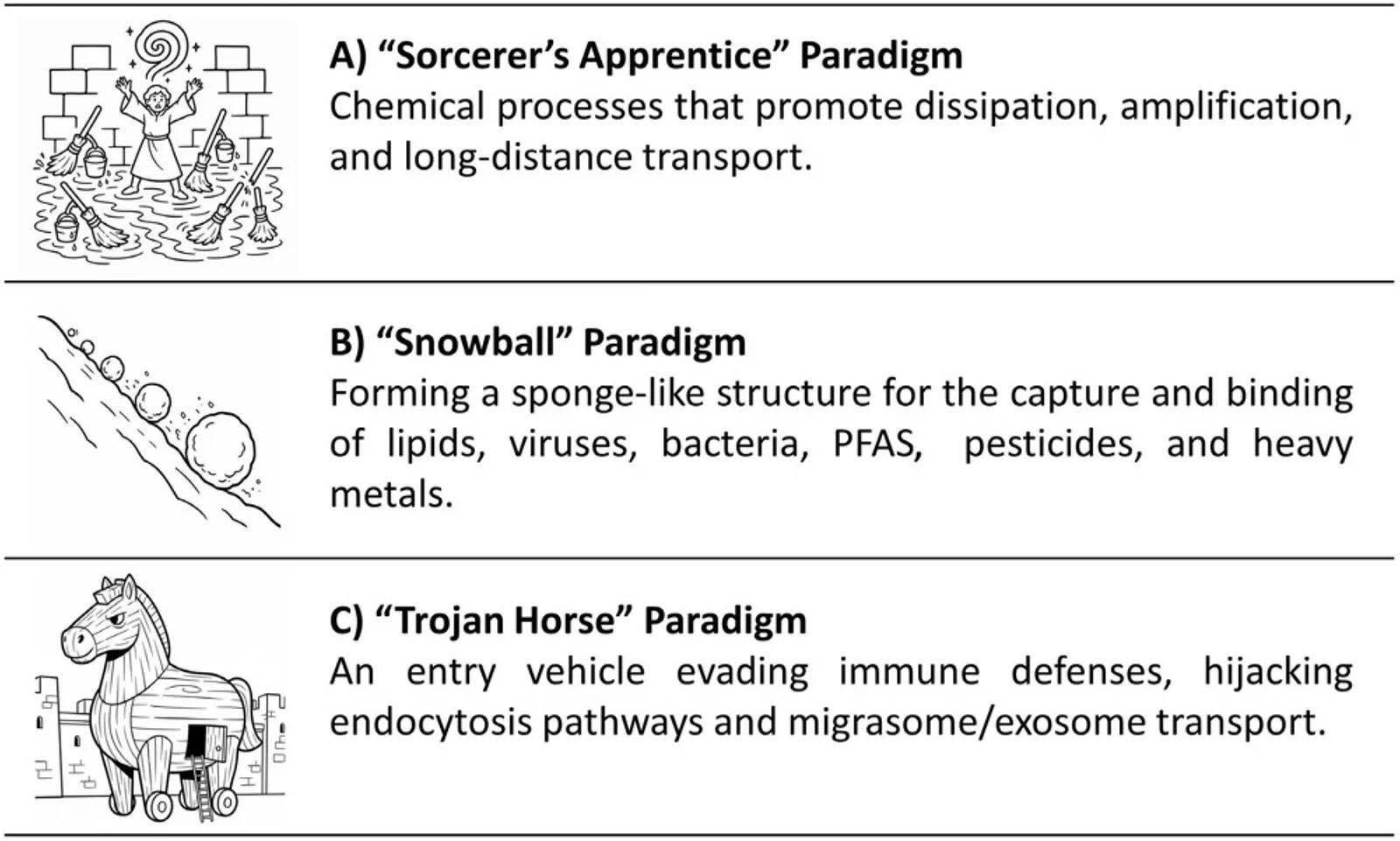
Proposed mechanisms by which MNPs may contribute to human disease. (A) “Sorcerer’s Apprentice”: physicochemical amplification and long-distance dissemination of pollutants. (B) “Snowball” paradigm: formation of sponge-like aggregates that adsorb lipids, PFAS, pesticides, heavy metals, and biological agents, increasing toxic burden. (C) “Trojan Horse”: cellular entry of MNP-associated cargo through evasion of immune defenses and hijacking of endocytic and extracellular vesicle pathways. Together, these mechanisms may explain the systemic biological effects of MNP exposure and support therapeutic removal strategies. The figure was generated using Leonardo AI.

## Data Availability

The datasets used and analyzed during the current study are available from the corresponding author upon reasonable request.
